# Laser-Directed Bubble Printing of MXene-Based Composites: A Simple Route to Micropatterned Photodetectors

**DOI:** 10.1021/acsami.5c08769

**Published:** 2025-06-23

**Authors:** Marcel Herber, Bianca M. Hanly, Eric H. Hill

**Affiliations:** † Institute of Physical Chemistry, 14915 University of Hamburg, Grindelallee 117, 20146 Hamburg, Germany; ‡ The Hamburg Center for Ultrafast Imaging (CUI), Luruper Chaussee 149, 22761 Hamburg, Germany

**Keywords:** directed assembly, nanoparticle assembly, laser printing, colloidal assembly, microbubbles, nanocomposites, perovskites

## Abstract

MXenes are a family of robust two-dimensional materials with tunable optical properties and high electrical conductivity ideal for optoelectronics. Herein, the direct, free-form micropatterning of MXene/semiconductor composites on glass substrates using a laser-driven microbubble is reported. As a proof-of-concept for the fabrication of photodetectors, this approach was used to print 0.09 mm^2^ square patches of Ti_3_C_2_T_X_ MXene combined with a number of different semiconductors. MXene composites printed with In_2_S_3_ at different ratios revealed that a 9:1 MXene:In_2_S_3_ ratio had increased responsivity compared to lower ratios or MXene alone. Out of the semiconductors studied, CsPbBr_3_ halide perovskite nanocrystals gave the highest responsivity value of 21.3 mA/cm^2^ under 369 nm illumination. Bubble printing of mixed colloids into micropatterned composite materials for photodetectors has great potential to advance sensing technologies across the electromagnetic spectrum while also requiring further optimization to enhance device performance. Overall, this further advances the application of MXenes in electronic and sensing devices, laying the foundation for future developments in patterning of miniaturized photodetectors.

## Graphical abstract

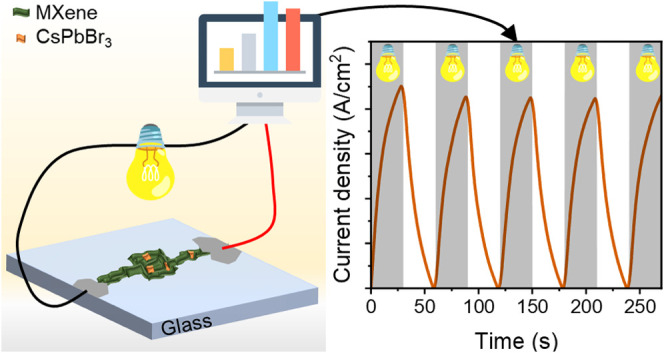

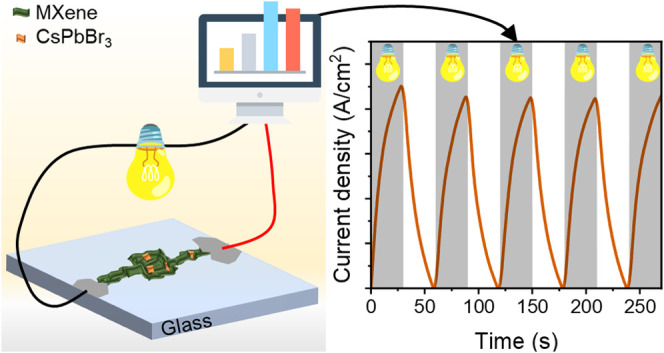

## Introduction

MXenes represent a recent family of two-dimensional materials first reported by Gogotsi and co-workers in 2011.^1^ MXenes can be broadly described by the formula M_*n*+1_X_*n*_T_X_, where M stands for a transition metal such as titanium, niobium or vanadium, X indicates carbon and/or nitrogen, and T is the type of surface termination (usually oxygen, hydroxyl or fluorine). In recent years there has been a growing interest in MXenes because of their remarkable properties. They exhibit tunable optical properties,^2^ electrical conductivity,^3^ high mechanical strength,^4^ biocompatibility,^5^ ionic conductivity and storage capacity,^6^ and can effectively block electromagnetic radiation in the microwave range.^7^ As a result, MXenes have found a wide range of applications across various fields, including optoelectronics,^8^ electromagnetic interference shielding,^7^ medical applications,^5^ energy storage solutions,^6^ memristors,^9^ and sensors.^10^

Photodetectors are devices that convert light into electrical signals by absorbing photons that generate charge carriers (electrons and holes), within a semiconductor.^11^ Photodetectors can operate over a range of wavelengths, from ultraviolet to infrared, and are classified into several types, including organic photodetectors,^12^ phototransistors,^13^ and photodiodes,^14^ each tailored for specific applications. Their ability to detect and quantify light makes them essential components in a wide range of technologies, including optical communications,^15^ photoimaging,^16^ medical diagnostics,^17^ and environmental sensing.^18^ Various techniques have been used to pattern photodetectors, including photolithography,^19^ electron-beam lithography,^20^ and nanoimprinting,^21^ each offering unique advantages such as resolution, scalability, and material compatibility.^22^

Utilizing the excellent electrical conductivity of MXenes, which enhances their capability to efficiently transport photogenerated charge carriers, MXene composites have also found use as photodetectors.^23,24^ While this has led to improvements in aspects like charge separation or stability,^25,26^ such photodetectors have so far taken the form of films or bulk composites. Recent advances in the micropatterning of MXenes, with spray coating^27^ and laser direct writing,^28^ have enabled the patterning of MXene-based photodetectors with robust performance. MXene composites such as Ti_3_C_2_T_X_ MXene-tellurium^29^ and Bi nanoparticles on Ti_3_C_2_T_X_,^24^ have shown high sensitivity, fast response times, and potential for integration into flexible and transparent optoelectronic devices.^29,30^ This underscores the versatility of MXenes as promising candidates for the next generation of photodetectors. Nevertheless, these methods have limitations including incompatibility with composite formation, sophisticated or expensive equipment, and the requirement for rheological additives to attain the desired printing characteristics.

Recently, the use of laser-driven microbubbles, or bubble printing (BP) to deposit colloidal matter at solid interfaces has emerged as a simple yet powerful approach for patterning with micron resolution. The heating of a colloid or plasmonic substrate with a laser results in the formation of a microbubble, and dispersed colloids are dragged toward the bubble via convection, where they are then deposited onto the substrate.^31^ A variety of colloids including polymers,^32^ quantum dots,^33^ metal nanoparticles,^34^ proteins,^35^ and clay nanomaterials^36,37^ have been deposited on solid substrates using BP. The heating of the solvent by a laser to form the microbubble is aided by the absorption of light by the colloid and its conversion into heat, which is highly efficient in MXenes,^38^ allowing their BP onto various substrates.^37,39^ In this context, BP emerges as a promising alternative, offering a simple colloidal approach for integrating MXenes in composite materials while avoiding the complexities associated with traditional techniques.

In this study, BP is demonstrated to be a straightforward, postprocessing-free method for the fabrication of MXene-semiconductor composites, resulting in micropatterned photodetectors with different photoresponse characteristics. While MXene exhibits a photoresponse alone, the incorporation of small amounts of other semiconductors such as CsPbBr_3_ nanocrystals was carried out, resulting in enhanced sensitivity. The dual functionality enabled by BP presents exciting opportunities for future developments in photodetector design, as it suggests a pathway to tailor devices for specific applications across different regions of the electromagnetic spectrum. However, optimizing materials and ratios is crucial for maximizing overall performance. Further modifications to the process which can leverage interactions between 2D materials and molecules at their interfaces may lead to enhanced performance and versatility in photodetectors, ultimately broadening the scope of this approach for use in advanced sensing technologies and applications in the fields of telecommunications, environmental monitoring, and biomedical diagnostics.

## Experimental Section

### Materials

Lithium fluoride (LiF, ≥99%) and thioacetamide (TAA, ≥99%) were obtained from Roth, titanium aluminum carbide 312 (Ti_3_AlC_2_, ≥90%, ≤40 μm), cetyltrimethylammonium bromide (CTAB, ≥99%), polyaniline (emeraldine base) (PANI, average *M*_w_ ∼ 50k) and poly­(3,4-ethylenedioxythiophene) polystyrenesulfonate (PEDOT:PSS, 3.0–4.0% aqueous dispersion) were obtained from Sigma-Aldrich, tetrahydrofuran (THF, ≥99.9%) was obtained from Chemsolute, Indium­(III) chloride tetrahydrate (InCl_3_·4 H_2_O, 99.99%) was obtained from J&K, hydrochloric acid (HCl, 37%) and isopropanol (IPA, ≥99.7%) were obtained from VWR Chemicals, and silver paste (Acheson 1415) was obtained from Plano GmbH. Milli-Q water with a resistivity of 18.2 MΩ cm was used for synthesis, cleaning of the product, and for rinsing of the prepared samples. Copper sulfide (CuS) nanoplatelets in toluene were synthesized by the protocol of Wu et al.^40^ CsPbBr_3_ nanocubes of 9 × 9 × 9 nm in hexane were synthesized by the protocol of Tong et al.,^41^ and their concentration was calculated following the approach described by Maes et al.^42^

### Ti_3_C_2_T_X_ MXene Synthesis

Ti_3_C_2_T_X_ nanosheets were synthesized by using the minimally intensive layer delamination method by Thakur et al.^43^ In a PTFE bottle containing 80 mL of 9 M hydrochloric acid, 6.4 g of lithium fluoride was added while stirring at 200 rpm for 5 min. Subsequently, 4 g of Ti_3_AlC_2_ MAX phase powder was gradually added over a period of 4 min. The stirring speed was then increased to 400 rpm. After 24 h, the resulting dark-black dispersion was centrifuged with Milli-Q water for washing (5 min per cycle at 3240 rcf) until a neutral pH was reached. The swollen MXene clay was redispersed in Milli-Q water and vortexed for 30 min, followed by sonication in an ice bath for 15 min under a nitrogen flow, and then centrifuged for 30 min at 2380 rcf. The collected supernatant exhibited a dark-green color and had a MXene concentration of 33.9 mg/mL. X-ray diffraction (XRD) analysis revealed the characteristic (00*l*) planes (Figure S1a), while the solution displayed a plasmon band at 741 nm (Figure S1b).

### In_2_S_3_ Synthesis

Hydrothermal synthesis was used to synthesize In_2_S_3_. First, 280 μL of a CTAB aqueous solution (0.1 M) and 5.22 mL of Milli-Q water were combined in a 50 mL Teflon beaker. Subsequently, 1.75 mmol of an InCl_3_ (7 mL, 0.25 M) solution was added and the mixture was stirred for 15 min. Next, 17.5 mL of TAA (0.8 M) was added and the Teflon beaker was sonicated for an additional 15 min. The molar ratio of InCl_3_ to TAA was maintained at 1:8. Finally, the reaction mixture was degassed with nitrogen for 10 min and the Teflon beaker was placed into an autoclave (DAB-2, Berghof Instruments GmbH, Germany).The autoclave was heated at 180 °C in a preheated oven while stirring for 15 h, then allowed to cool to room temperature. The resulting product was washed four times with water and three times with ethanol, with each washing followed by centrifugation at 5350 rcf for 15 min. Finally, the product was dried in a vacuum oven at 60 °C for 24 h.

### Bubble Printing

MXene-In_2_S_3_ dispersion containing 25% isopropanol in the ratios 1:0, 1:1, 2:1, 4:1 and 9:1 of Ti_3_C_2_T_X_ MXene (33.9 mg/mL) and In_2_S_3_ (33.9 mg/mL) were prepared. For this, 10 μL MXene or MXene-In_2_S_3_ were mixed with 110 μL Milli-Q water and 40 μL isopropanol. For the preparation of MXene-PANI in the ratio 9:1, 13.3 μL Ti_3_C_2_T_X_ MXene (33.9 mg/mL) were mixed with 13.3 μL PANI in THF (3.77 mg/mL), 40 μL isopropanol and 93.4 μL Milli-Q water, and for MXene-PEDOT:PSS in the ratio 9:1, 13.3 μL Ti_3_C_2_T_X_ MXene (33.9 mg/mL) were mixed with 5 μL PEDOT:PSS (10.0 mg/mL), 40 μL isopropanol and 101.7 μL Milli-Q water. To prepare MXene-CuS and MXene-CsPbBr_3_, 200 μL of Ti_3_C_2_T_X_ MXene (33.9 mg/mL), 200 μL Milli-Q water and 200 μL of CuS in toluene (25.3 mg/mL) or CsPbBr_3_ cubes in hexane (2.53 μM, 5.26 mg/mL) were stirred overnight for a phase transition approach. Afterward, the aqueous phase was separated and 30 μL of each was mixed with 10 μL isopropanol. The mixtures were subjected to sonication and vortexing for 5 s. A 0.12 mm imaging spacer (SecureSeal, Grace Bio-Laboratories, USA) was firmly adhered to a glass coverslip (Thickness 1). Subsequently, 15 μL of the prepared MXene or MXene-composite dispersion was pipetted into the well, which was subsequently covered with a glass coverslip. The substrate was then placed on the stage of an inverted microscope. A 532 nm continuous wave laser (Torus 532, Laser Quantum, UK) was directed through a 5× beam expander (GBE05-A, Thorlabs, USA) before entering the microscope (Eclipse Ti2-A, Nikon, Japan). The laser was reflected by a 532 nm dichroic beam splitter and focused onto the interface between the substrate and the dispersion, achieving a spot size of approximately 0.7 μm, using a 10× air objective (numerical aperture = 0.45, CFI Plan Apochromat Lambda D 10X, Nikon). The sample was imaged under transmitted light with a CCD camera through a 533 nm notch filter (NF533-17, Thorlabs, USA) to block the laser light. A motorized microscope stage (H117P1NN, Prior Scientific Instruments, UK) facilitated the movement of the laser beam. The samples were printed using an optical chopper system (MC2000B-EC, Thorlabs, USA) with a chopper blade (MC1F10HP, Thorlabs, USA) operating at 5000 Hz and a laser power of 49.5 mW. MXene-CuS was printed without using a beam-chopper. After the printing process, the top coverslip was removed, and the substrate was rinsed with Milli-Q water and briefly dried with compressed air. A scheme of the optical setup is shown in Figure S2. For every MXene or MXene composite 2 samples were bubble-printed and tested.

### Characterization

For resistance measurements, MXene or MXene-composite patterns prepared by BP were connected to an electrochemical workstation (Zahner Zennium XC, Zahner-Elektrik GmbH & Co. KG, Germany) after contacting with silver paste. The current–voltage characteristics of the samples were measured over a range of −5 to 5 V. The same electrochemical workstation was used for photocurrent measurements. The bubble-printed patterns on glass substrates contacted with silver paste were placed in a sample holder and connected to the electrochemical workstation. Chopped light voltammetry was performed with an applied bias of 5 V, utilizing light pulses with a duration of 30 s. The illumination sources were LEDs emitting white light, 369 nm, 453 and 538 nm (LSW-1, LS365-2, LS447 and LS530, Zahner-Elektrik GmbH & Co. KG, Germany). The emission spectrum for the white light source (LSW-1) is given in Figure S3. UV/vis spectroscopy was done on a Cary 60 (Agilent Technologies, USA). X-ray diffraction (XRD) patterns were measured with Cu–K_α_ radiation (1.54 Å) on a PANalytical X’Pert MPD diffractometer (Philips, Netherlands). To obtain transmission electron microscopy (TEM) images a JEOL JEM 1011 (JEOL, Japan) with an acceleration voltage of 100 kV was used, while for scanning electron microscopy (SEM) images, and electron-dispersive X-ray spectroscopy (EDX) of the bubble-printed lines a Zeiss LEO Gemini 1550 with an acceleration voltage of 2 kV (Carl Zeiss Microscopy Deutschland GmbH, Germany) was used. Raman spectra were measured using an inverted microscope with a 60× objective (NA = 0.85, CFI Plan Fluor, Nikon) and a 785 nm CW laser (LM-785-PLR-175, Ondax, USA) with an optical power of 2.6 mW. A 785 nm long-pass filter (RazorEdge, Semrock, USA) was used to filter the scattered light and the light passed through a 10 μm slit. This light was then reflected off of a 770 nm blaze, 500 lines/mm grating (Richardson Gratings, Newport Spectra-Physics GmbH, Germany), in a spectrograph (Kymera 328i, Andor Technology, U.K.) and detected using a back-illuminated CCD camera with a sensor temperature of −80 °C (Newton 970, Andor). Raman signal in the range of 242–2112 cm^–1^ was collected and integrated over 60 s. For height measurements, printed lines were measured with an atomic force microscope (JPK NanoWizard 4XP-, Bruker, USA) with a PPP-NCHR tip with a tip diameter of 10 nm (Nanosensors, Switzerland). AFM measurements were recorded in Quantitative Imaging mode at a force of approximately 280–350 nN.

## Results and Discussion

Briefly, Ti_3_C_2_T_X_ MXene was synthesized by selectively etching away the aluminum layer from the Ti_3_AlC_2_ MAX phase through the in situ generation of hydrofluoric acid in a solution of lithium fluoride and hydrochloric acid.^43^ The resulting Ti_3_C_2_T_X_ MXene shows typical flake-like morphology and a distinct plasmon band at 741 nm (Figure S1c). The procedure for generating MXene composite-based patterns with BP is illustrated in Figure 1a and can be summarized as follows: (1) 15 μL of MXene composite dispersion is pipetted onto a glass substrate in the well created by an imaging spacer. (2) The laser is focused at the interface between the substrate and the dispersion, where the absorption of light by the MXene nanosheets generates heat, leading to the formation of a microbubble at the substrate–liquid interface.^38^ The surface tension gradient resulting from the presence of the bubble drives Marangoni convection, which draws colloidal particles toward the microbubble. (3) Upon reaching the bubble interface, particles become fixed onto the substrate at the triple contact line due to various forces, particularly capillary and van der Waals interactions. The movable stage of the microscope allows for the printing of patterns with the deposited colloidal particles. (4) Finally, the bubble-printed MXene/composite patterns on the glass substrate are rinsed with water, dried under compressed air, and contacted on the edges of the substrates with silver paste for testing.

**Figure 1:**
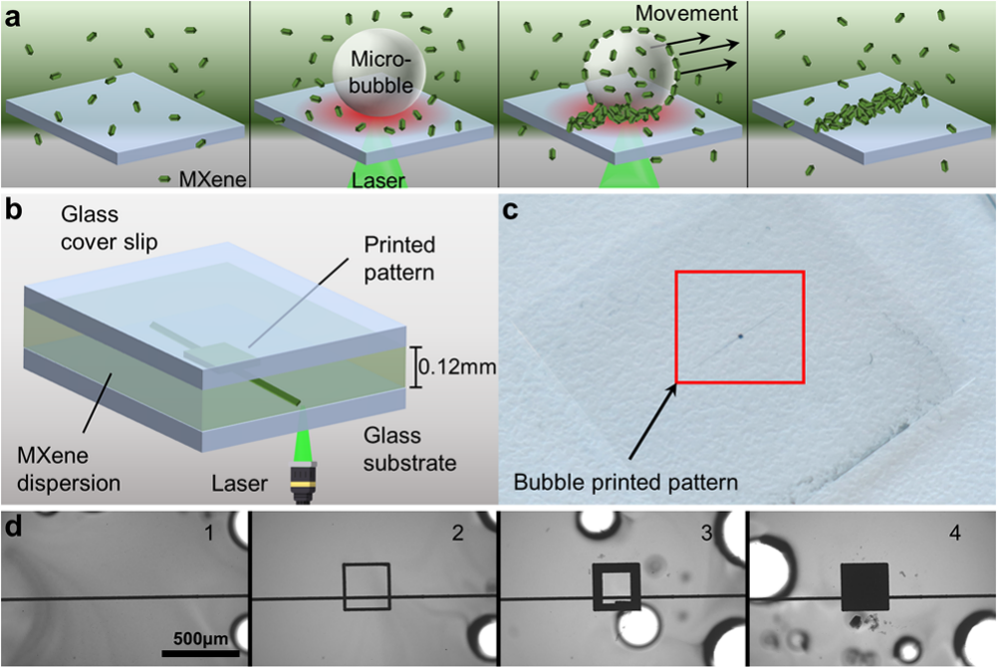
(a) Schematic of BP; (b) schematic of bubble printing MXene particles between a glass substrate and a glass coverslip to create patterns of MXene; (c) photograph of the printed pattern; (d) microscope images of bubble printing of a MXene pattern: (1) printing a horizontal line, (2) printing the outer lines, (3) filling the square, and (4) final printed pattern. A video corresponding to (d) is shown in Video S1.

BP was carried out on an inverted optical microscope which focuses a continuous wave laser at the interface between a glass substrate and the colloidal dispersion of the MXene composite (Figures 1 and S2). A typical printed pattern with a size of 300 × 300 μm was created, in which the area of 0.09 mm^2^ is visible to the naked eye (Figure 1c). The square patterns were formed by first bubble printing a long horizontal line, followed by bubble printing the outline of the square shape, and then filling in the square by moving the microscope stage in a spiral with a slight overlap of 5–10 μm between lines (Figure 1d).

Herein the capability to print on glass surfaces without the need for surface functionalization was demonstrated, representing an advance compared to our initial findings, in which APTES (3-aminopropyltriethoxysilane) modification was needed to achieve good adhesion of the MXenes onto glass.^39^ This is attributed to the batch-to-batch variability in surface chemistry of MXene, leading to distinct surface properties such as roughness, surface energy, and functional group density.^44,45^ In particular, the ratio of surface functional groups (−OH, −COOH, −F) can significantly alter substrate adhesion. This finding underlines the importance of understanding the surface chemistry of MXenes in order to enhance the efficacy of printing techniques and ensure reproducibility.

Composites of MXene with In_2_S_3_ at different ratios were studied, due to their relatively similar edge lengths and sheet-like structure (Figure S4a,b) promoting intercalation of the sheets during printing. In the process of fabricating MXene-In_2_S_3_ composites, it was observed that increasing the ratio of In_2_S_3_ to MXene resulted in an unstable colloidal dispersion, where 2:1 In_2_S_3_ to MXene and above led to sedimentation. This is attributed to the reduced dispersibility of the In_2_S_3_ particles in water comparison to MXene. Ensuring a ratio of MXene and composite which provides good colloidal stability is therefore crucial for attaining the desired printing behavior of the mixture.

Scanning electron microscopy (SEM) and energy dispersive X-ray spectroscopy (EDX) were performed to investigate the morphological and elemental characteristics of various MXene composite samples. SEM revealed distinct variations in surface morphology depending on MXene:semiconductor ratio and the semiconductor used (Figure 2). The overview of the entire 300 × 300 μm^2^ pattern shows that MXene and MXene-In_2_S_3_ patterns (Figures 2a,b, and S5) exhibit thinner line widths and smoother surfaces compared to MXene-CuS and MXene-CsPbBr_3_ (Figure 2c,d), which exhibit inconsistent thicknesses within the pattern. At higher magnifications, no significant morphological differences are observed between MXene and MXene-In_2_S_3_ patterns; however, EDX analysis confirms the presence of In and S in MXene-In_2_S_3_, with a corresponding increase in their relative abundance with increasing In_2_S_3_ (Figure S6a–c). The micropatterned composite of MXene with CuS nanoplates exhibits aggregated particles dispersed throughout the substrate (Figure 2c), with EDX confirming the presence of Cu and S (Figure S6d). In contrast, the composite of MXene with CsPbBr_3_ nanocubes has a relatively uniform distribution of particles with minimal aggregation (Figure 2d), with the presence of Cs, Pb, and Br confirmed by EDX (Figure S6e). Interestingly, the MXene-CsPbBr_3_ micropatterns have fewer large pores at the interface compared to the pure MXene and other composite micropatterns. The elemental analysis results from EDX for each sample are given in Table S1 in the Supporting Information, while EDX spectra are given in Figure S6.

**Figure 2:**
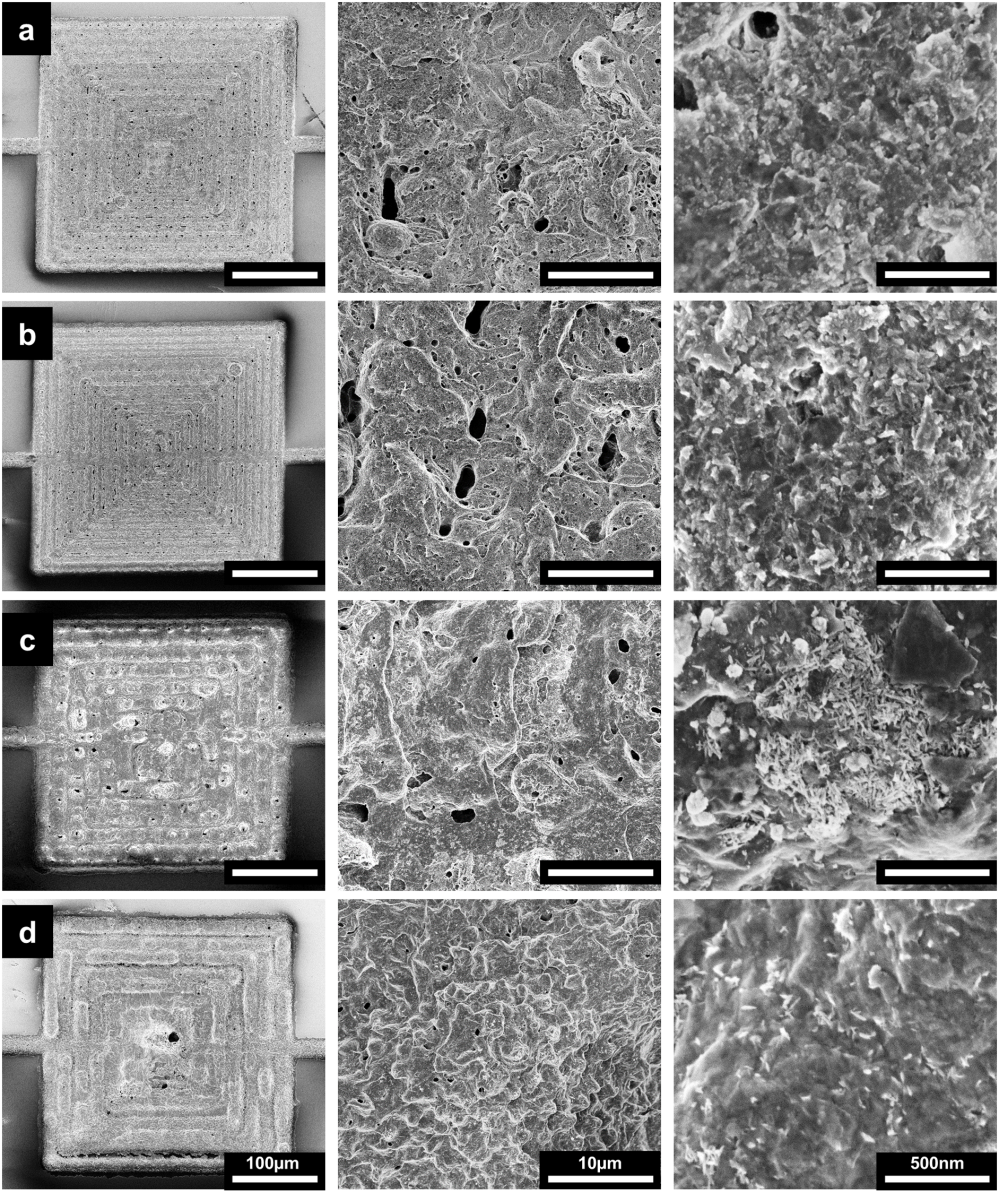
Scanning electron micrographs of bubble-printed (a) MXene, (b) 9:1 MXene-In_2_S_3_, (c) MXene-CuS, and (d) MXene-CsPbBr_3_. Scale bars are left 100 μm, middle 10 μm, and right 500 nm.

Atomic force microscopy (AFM) measurements were performed to investigate the surface morphology in more detail (Figure S7). The AFM images reveal distinct linear features corresponding to the printed lines, where MXene and 9:1 MXene:In_2_S_3_ (Figure S7a,b) exhibit thinner lines, while the other composites show broader structures. Furthermore, MXene-CuS (17.6 μm) and MXene-CsPbBr_3_ (17.7 μm) (Figure S7c,f) have substantially increased height compared to the rest of the composites, particularly pure MXene (7.1 μm). The uniformity of the height of the printed structures varies between composites, and some voids between printed lines can be observed.

### Influence of MXene-In_2_S_3_ Ratio

The current–voltage (*IV*) characteristics of the bubble-printed patterns were measured in the dark to compare the influence of material composition (Figure S8a). The *IV* curves exhibited a linear response within the range of – 5 to 5 V at all compositions, indicating ohmic conductivity, with a resistance of 5.12 kΩ up to 9.29 kΩ. The photocurrent density of MXene-In_2_S_3_ composites was evaluated using chopped light voltammetry with different light sources (369 nm, 453 nm, 538 nm and white light), revealing a dependence of the measured signal on the amount of In_2_S_3_ in the dispersion during printing (Figures 3a and S9). The measured photocurrents reveal significant differences between bubble-printed patterns with different ratios of MXene:In_2_S_3_. The MXene:In_2_S_3_ ratio of 9:1 consistently demonstrated the highest photocurrent densities, approximately twice as much as pristine MXene and 4:1 MXene:In_2_S_3_, and nearly four times greater than 2:1 MXene:In_2_S_3_ and 1:1 MXene:In_2_S_3_. For example, under illumination at 369 nm (2400 W/m^2^), 9:1 MXene:In_2_S_3_ shows a photocurrent density of 1.8 mA/cm^2^, as opposed to 0.49 mA/cm^2^ for the 2:1 composite. This indicates that a 9:1 ratio of MXene to In_2_S_3_ composite provides a balance between photocurrent generation and electronic conductivity. Notably, the relatively high performance of the 9:1 composite is consistently observed across different light sources, underscoring the robustness of 9:1 MXene-In_2_S_3_ under varied illumination conditions (Figures 3a and S9).

**Figure 3:**
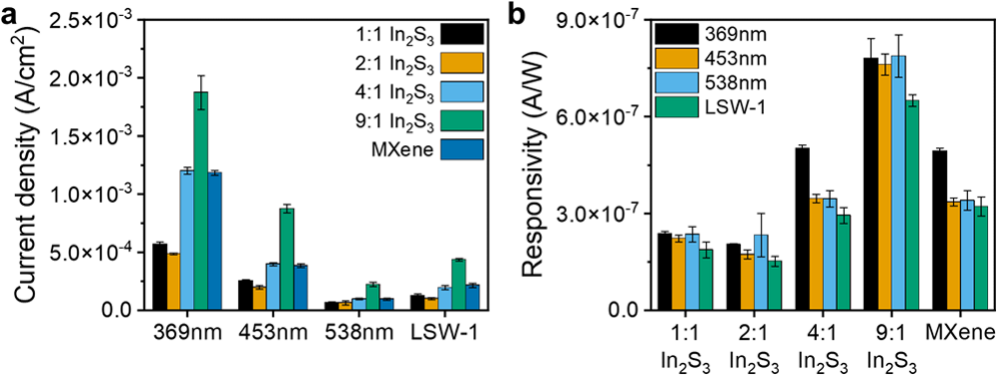
(a) Photocurrent density for different MXene-In_2_S_3_ ratios and different light sources with an intensity of 100%; (b) responsivity for different MXene-In_2_S_3_ ratios and different light sources. The individual photoresponse curves for (a) and (b) are given in Figures S9 and S11, respectively, and photocurrent densities at different light powers are given in Figure S10.

In order to systematically compare the performance of different samples as photodetectors under different light sources, the figure of merit responsivity (A/W) was used as the primary metric for evaluation (Figures 3b and S11). 9:1 MXene:In_2_S_3_ had the highest responsivity, followed by pure MXene and 4:1 MXene:In_2_S_3_ (Figure 3b). In contrast, 2:1 and 1:1 MXene:In_2_S_3_ exhibit the lowest responsivity values across all measured light sources. This trend is consistently observed, where 9:1 MXene:In_2_S_3_ shows consistent responsivity values (0.76–0.79 μA/W) at wavelengths of 369, 453, and 538 nm, indicating effective light absorption and carrier generation within these spectral regions. However, there is a noticeable decrease in responsivity under broadband (400–750 nm, Figure S3) white light illumination (0.65 μA/W), suggesting limitations in detecting longer wavelengths with these composites. Similar trends are observed for 1:1 MXene:In_2_S_3_ (0.22–0.24 μA/W) and 2:1 MXene:In_2_S_3_ (0.17–0.23 μA/W) at wavelengths of 369, 453, and 538 nm, both of which exhibit almost 3.6 times lower responsivity compared to 9:1 MXene:In_2_S_3_. Also, pure MXene (0.34 μA/W) and 4:1 MXene:In_2_S_3_ (0.35 μA/W) show the same trend at wavelengths of 453 nm, and 538 nm with an almost 2.2 times lower responsivity than 9:1 MXene:In_2_S_3_. On the other hand, pure MXene and 4:1 MXene:In_2_S_3_ show a higher responsivity under 369 nm light, around 0.49 μA/W, which indicate an increased sensitivity to ultraviolet light.

The observed differences in responsivity can be attributed to several factors influenced by the ratio of MXene to In_2_S_3_ in the MXene-In_2_S_3_ composites. The optimal ratio between MXene and In_2_S_3_ can enhance the effective charge separation and transport mechanisms that are critical for photodetection.^46^ MXene acts as an electron acceptor, facilitating the extraction of photoexcited electrons from In_2_S_3_ and their rapid transport due to its high conductivity, reduces recombination losses within In_2_S_3_ and improves the photoresponse characteristics of the device.^47^ With a 9:1 ratio of MXene to In_2_S_3_, there is likely a continuous conductive network due to the large amount of MXene in relation to In_2_S_3_. The observed photoresponse in MXene-In_2_S_3_ composites at a 9:1 ratio can be attributed to several synergistic factors analogous to those described in substitutional doping in nanocrystal superlattices.^48^ This composition has a continuous conductive network within the composite, leveraging the high electrical conductivity of MXene while providing charge carrier generation by In_2_S_3_. In contrast, the high amount of In_2_S_3_ in the 1:1 and 2:1 composites results in reduced electrical conductivity, and also can potentially lead to increased carrier recombination, resulting in lower overall responsivity. These mechanisms underscore the importance of nanoscale interactions in engineering advanced composite materials, which should be strongly considered in future approaches.

Apart from responsivity (*R*), another important figure of merit for photodetectors is Noise-Equivalent-Power (*NEP*), which indicates the smallest incident power that the detector can differentiate a signal from the background noise and can be approximated as $\mathrm{NEP}=\frac{P_{\mathrm{in}}\times I_{\mathrm{noise}}\times A}{I_{\mathrm{ph}}}$ , where *P*_in_ is the incident optical power, *I*_noise_ is the noise current, *A* is the active surface area, and *I*_ph_ is the generated photocurrent. Additionally, specific detectivity (D*), defined as $D^{*}=\frac{\sqrt{A}\times \sqrt{\Delta f}}{\mathrm{NEP}}$ , where *q* represents the electron charge, and Δ*f* the electrical bandwidth.^49^ Table S2 compares the figures of merit of the different MXene-In_2_S_3_ composites and different light sources. Among these, 9:1 MXene:In_2_S_3_ showed the highest D* with 41.2 × 10^2^ Jones and a *NEP* of 36.3 W at 538 nm. Notably, as the wavelength shifts from visible to the UV, the D* for 9:1 and 1:1 MXene-In_2_S_3_ composites remains consistent, e.g. 41.0 × 10^2^ Jones for 9:1 MXene:In_2_S_3_ at 369 nm. These results support that, among the MXene: In_2_S_3_ composites studied herein, a ratio of 9:1 MXene:In_2_S_3_ is the optimal composition for bubble-printed photodetectors, and shows that the ratio of the two nanomaterials in the composite can significantly impact the overall performance of photodetectors. However, the overall increase in responsivity was poor compared to MXene alone, and thus, a broader comparison of bubble-printed MXene-based composites fabricated with other semiconductors was carried out.

### Comparison of MXene-Semiconductor Composites

The optimization of MXene-In_2_S_3_ composites highlights the pivotal role of material composition in determining electrical characteristics. However, it is also crucial to assess a diverse range of materials to demonstrate the generalizability of this approach. The unique electrical properties, charge carrier dynamics, and interactions exhibited by different materials can significantly impact the performance of MXene-based composites as photodetectors. Thus, CuS nanosheets, CsPbBr_3_ nanocubes, and polymers PANI (polyaniline) and PEDOT:PSS (poly­(3,4-ethylenedioxythiophene) polystyrenesulfonate) were printed with MXenes, providing insight into further enhancement of composite performance. To assess the impact of material, first, the *IV* characteristics of the bubble-printed patterns were evaluated in the dark (Figure S8b). The *IV* curves exhibited a linear response within the voltage range of – 5 to 5 V for all compositions, indicating ohmic conductivity, with resistances ranging from 11.3 kΩ to 60.5 kΩ. This shows that all composites have suitable *IV* characteristics for use in devices.

The photocurrent density of the composites was compared with different light sources as described in the previous section, revealing a strong influence of the semiconductor incorporated into the MXene composite on the measured photocurrent (Figures 4a and S12). Notably, MXene-CsPbBr_3_ consistently demonstrated the highest photocurrent densities, ∼18 times as much as pristine MXene, and ∼ 2.5 times greater than MXene-CuS, when illuminated at 369 nm. For example, under illumination at 369 nm (2400 W/m^2^) MXene-CsPbBr_3_ and MXene-CuS exhibited photocurrent densities of 21.3 mA/cm^2^ and 8.78 mA/cm^2^, respectively. Under all visible light sources MXene-CsPbBr_3_ exhibited the most significant photocurrent density, followed by MXene-PANI and MXene-CuS, underscoring the sensitivity and performance of MXene-CsPbBr_3_ across the visible spectrum (Figures 4a and S12).

**Figure 4:**
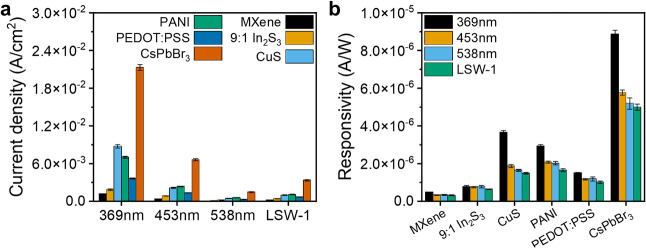
(a) Photocurrent density for different MXene composites and different light sources with an intensity of 100%; (b) responsivity for different MXene composites and different light sources. The individual spectra for (a) and (b) are given in Figures S12 and S14, respectively, and photocurrent densities at different light powers are given in Figure S13.

The different bubble-printed composite photodetectors were compared using the figure of merit responsivity (A/W) as the primary metric for evaluation (Figures 4b and S14). MXene-CsPbBr_3_ exhibited the highest overall responsivity, followed by MXene-PANI and MXene-CuS. Conversely, 9:1 MXene:In_2_S_3_ and pure MXene exhibited the lowest responsivity values across all measured light sources (Figure S14). The highest responsivity values of 8.88 μA/W at 369 nm, 5.77 μA/W at 453 nm, 5.20 μA/W at 538 nm, and 5.00 μA/W for broadband white light were obtained for MXene-CsPbBr_3_. However, a discernible decline in responsivity is evident at longer wavelengths, correlating well with the absorption spectrum of CsPbBr_3_ cubes (Figure S1f). A similar trend is observed for MXene-CuS with values of 3.66 μA/W at 369 nm, 1.88 μA/W at 453 nm, 1.65 μA/W at 538 nm, and 1.49 μA/W for broadband white light. However, no significant difference is noted between 453 and 538 nm for MXene-PANI and MXene-PEDOT:PSS, and it is expected that their responsivity increases with the overlap with their absorption maxima at 583 nm and >600 nm (Figure S1d,e). All MXene composites demonstrated greater responsivity under 369 nm irradiation than under visible light.

The observed differences in responsivity among MXene composites can be attributed to the choice of semiconducting material. For instance, CsPbBr_3_ demonstrates superior optoelectronic properties compared to CuS due to higher charge carrier mobility and enhanced photonic absorption characteristics.^50^ Recent studies have shown that the formation of a heterojunction between CsPbBr_3_ and Ti_3_C_2_T_X_ MXene facilitates efficient charge separation. It was demonstrated that in the MXene-CsPbBr_3_ system favorable band alignment at the interface supports charge separation, thereby promoting photogenerated electron transfer from the perovskite to the MXene, resulting in enhanced photocurrent and faster photoresponse (see energy-band diagrams in Figure S15).^51,52^ Furthermore, drift-diffusion modeling has shown that incorporation of MXenes into the CsPbBr_3_ layer of perovskite solar cells leads to enhanced collection of holes from the hole transport layer due to favorable band alignment.^53^ CuS exhibits good electrical conductivity and a suitable band gap of 2.5 eV,^54^ however, it has lower charge transport efficiency and higher rates of carrier recombination compared to CsPbBr_3_. The superior performance of CsPbBr_3_ is attributed to its balanced electron and hole mobility, defect tolerance, and high-quality crystal structure, which contribute to its enhanced effectiveness in these aspects relative to CuS.^55,56^ However, in such systems, the measured photocurrent may result from a combination of photoconductive transport, photovoltaic junction effects, and photothermal contributions. This is particularly relevant for MXene, due to its high light-to-heat conversion efficiency. In contrast to CsPbBr_3_ and CuS, the MXene-In_2_S_3_ interface exhibits a smaller conduction band offset, and charge separation may be predominantly drift-driven under applied bias, with additional contributions from trap states within the In_2_S_3_ phase.^57^ Although In_2_S_3_ has a suitable bandgap, it exhibits limitations in charge mobility and may form less favorable interfaces with MXene, resulting in reduced responsivity in the composite. These findings highlight the critical role of the second material in optimizing the performance of MXene composites, with factors such as charge carrier mobility, band gap alignment, and interface quality significantly influencing responsivity.

A comparative analysis of the figures of merit for different MXene composites and different light sources was carried out (Table S3). MXene-CsPbBr_3_ composites demonstrated the highest D* of 99.1 × 10^3^ Jones and a *NEP* of 1.27 W at 369 nm. As the excitation shifts to longer wavelengths, the D* for several MXene composites decreases, with 58.0 × 10^3^ Jones for MXene-CsPbBr_3_ at 538 nm. These findings show that the composite of MXene and CsPbBr_3_ led to the best performance out of the tested materials, and demonstrate that the material in the composite can significantly impact the overall performance of photodetectors. However, it is expected that the specific detectivity of MXene-CsPbBr_3_ composites can be further improved by dispersion of both materials in miscible solvents, which was not the case herein.

In order to place our results in the broader context of the field of MXene composites for photodetection, it is important to first note that the majority of studies use large-area fabrication techniques, such as spin coating or spray coating, which have yielded remarkable figures of merit. For example, Ti_3_C_2_ MXene/Silicon with a responsivity of 402 mA/W and a D* of 2.03 × 10^13^ Jones,^27^ Ti_3_C_2_T_X_ MXene/CsPbBr_3_ with a responsivity of 44.9 mA/W and a D* of 6.4 × 10^8^ Jones,^58^ and Ti_3_C_2_ MXene/ZnO showing a responsivity of 142.2 mA/W and a D* of 2.03 × 10^10^ Jones.^59^ In these applications, MXene is typically utilized as an electrode material. However, MXene itself also has some intrinsic responsivity, and the contribution of the MXene to the overall photodetector performance cannot be ruled out.

The inherent responsivity of MXene contributes to the overall performance of the photodetector, enhancing its ability to convert light into detectable electrical signals efficiently.^11^ IMPS (intensity modulated photocurrent spectroscopy) measurements were used to characterize the response to light at different frequencies, revealing a stronger response at higher frequencies (Figure S16). This correlates with previous time-resolved characterization of MXene-based photodetectors showing a photoresponse on the 0.1 to 1 ns time scale.^23^ The intrinsic responsivity of MXene in photodetector applications is likely be due in part to its exceptional light-to-heat conversion efficiency (approximately 100%).^38^ This suggests that MXenes can function as thermal-type photodetectors through the photothermoelectric (PTE) effect.^11^ In essence, the PTE effect is the process by which the electron temperature within the material rises when exposed to light, resulting in a localized photothermal voltage generated via the Seebeck effect. Furthermore, such thermal-type photodetectors show promise for applications in detection of other wavelengths further in the infrared spectrum, which may benefit from this approach. MXenes, including Ti_3_C_2_T_*x*_, have been shown to exhibit photothermoelectric behavior with characteristic response times on the order of several seconds, as a result of thermal diffusion and carrier equilibration dynamics, which supports the continuous rise in photocurrent observed under illumination.^60^

Ensuring the long-term stability of MXene-based devices remains a critical challenge. MXenes are known to undergo oxidative degradation over time, which has been shown to negatively impact their structural integrity and electrical performance. This degradation is primarily attributable to the oxidation of surface terminations upon exposure to oxygen or moisture, resulting in the transformation of the metallic MXene into insulating oxide phases.^61–63^ Raman spectroscopy conducted on drop casted MXene films and the bubble-printed MXene patterns (Figure S1b) did not reveal any significant peak shifts that could arise from oxidation of the MXene or changes in stacking.^64^ To assess the stability of the bubble-printed MXene composites, their electrical properties and optical response were measured after a 62-day period in ambient air. The electrical resistance of the devices increased by a factor of 1.8 to 6.1 during this time (Figure 5a), although linear *IV* characteristics were preserved (Figure S17), suggesting that percolation pathways remained intact. In addition to the rise in resistance, a significant decrease in photoresponsivity was recorded (Figure 5b): after 62 days, pure MXene devices retained 15% of their original responsivity, 9:1 MXene:In_2_S_3_ composites 8%, MXene-CuS 6%, MXene-PANI 15%, MXene-PEDOT:PSS 25%, and MXene-CsPbBr_3_ 10%.

**Figure 5:**
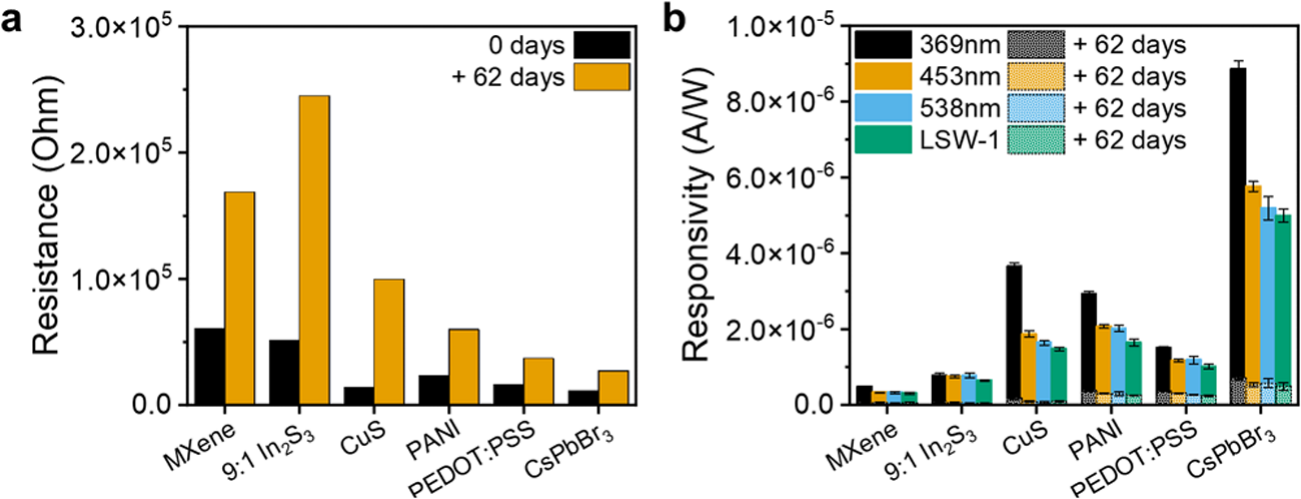
Stability of samples exposure to air. Comparison of sample (a) resistance and (b) responsivity before and after 62 days in ambient air. Corresponding spectra of photocurrent density responses are given in Figure S18.

Although the devices retained some response to light after prolonged exposure to air, these findings underscore the critical need for strategies to ensure long-term operational stability for real-world applications, especially in humid or oxidizing environments.^65–67^ For instance, hydrogen annealing has been demonstrated to not only restore the conductivity of oxidized Ti_3_C_2_ MXene films but also significantly retard further oxidation, even in humid and oxidizing environments.^66^ Interface engineering through chemical functionalization, such as introducing catechol-functionalized polymers or poly­(tannic acid) bridging molecules, has demonstrated pronounced improvements in both oxidation resistance and long-term conductivity for MXene-based films and hydrogels without compromising intrinsic electrochemical properties.^65,67^ Incorporating polymer encapsulation or surface modifiers that form hydrogen/coordination bonds with the MXene can provide physical and chemical barriers to environmental degradation, and such strategies can be integrated with encapsulation approaches such as atomic layer deposition of metal oxides or multilayer polymer coatings to provide multimodal protection.^68^

## Conclusions

The present study successfully demonstrated that the bubble printing technique can be used to fabricate MXene-semiconductor composite micropatterns in a single process. The resulting 0.09 mm^2^ patches were capable of detecting UV and visible light, and the chosen semiconductor material alongside MXene played a crucial role in device performance. The ratio of MXene to the second material in the composite was shown to be critical when aiming to attain optimal performance of the device. While the figures of merit of the bubble-printed photodetectors reported herein fall short of the state-of-the-art for photodetectors, this study provides strong support for BP as a rapid and simple approach to pattern functional nanomaterial composites on solid substrates. The key limitations of this approach include the presence of voids in the printed structures and the degradation of MXene under ambient conditions over time. These limitations can be addressed in future studies by implementation of strategies such as ink formulation, surface modification, and pattern encapsulation, which are anticipated to enhance charge transport, long-term stability, and the overall performance of the devices. Furthermore, optimization of material selection, ratios of MXene to semiconductor, and tuning of colloidal stability could open the door to bubble-printing of composites for photodetectors across different regions of the electromagnetic spectrum, and push toward applications beyond photodetectors.

There are several potential avenues to address the challenge of limited sensitivity of these MXene-based photodetectors, including capillary infiltration of conductive fillers after printing, which could improve interflake contact, reduce resistance, and enhance charge transport. Additionally, ink optimization through the controlled adjustment of viscosity, surface tension, and flake dispersion offers a direct strategy to adjust wetting behavior and structural uniformity during BP. In this study smaller MXene flakes were used, however, larger flake sizes can enhance conductivity by reducing the number of interflake junctions, thereby improving charge transport.^69^ The bubble printing of such functional composites is highly dependent on the compatibility of targeted light-sensitive materials/molecules with the solvents and colloidal conditions used, and their capability to form a stable colloidal dispersion. Due to the large number of high-quality semiconductor nanocrystals which are synthesized through hot-injection and heat-up syntheses in organic solvents, it would be beneficial for future efforts to disperse MXenes in aromatic or polar aprotic solvents miscible with solvents such as toluene and hexane. Although MXenes synthesized by HF etching have been previously dispersed in solvents such as *N*,*N*-dimethylformamide and *N*-methyl-2-pyrrolidone, MXenes fabricated through the minimally intensive layer delamination process used herein lack sufficient – F terminal groups for effective dispersion in these solvents. Surface modification of MXene to promote favorable interactions between MXene and semiconductor could lead to improved device sensitivity.

Overall, this work demonstrates the capability to leverage the exceptional electronic properties of MXene-based composites, and underscores the significant advantages of BP as a simple and fast colloidal method for optical patterning of multifunctional composite materials on solid substrates which does not require pre- or postprocessing steps. The ease with which various colloids, as well as polymers and small molecules, can be combined further highlights the potential of BP for fabrication of composites for photodetector applications across different electromagnetic domains.^11^ Moreover, BP of MXenes has been shown to be compatible with flexible polymeric substrates,^37^ showing great potential for development of flexible multifunctional optoelectronic devices. This work opens the door to future development of BP toward low-cost, miniaturized optoelectronic devices where substrate adaptability, custom patterning, and simultaneous deposition of colloidal species are essential.

## Supplementary Material





## Notes

XRD spectra of Ti_3_C_2_T_X_, In_2_S_3_ & CsPbBr_3_ cubes; Raman spectra of drop casted and bubble-printed MXene; UV–vis spectra of Ti_3_C_2_T_X_, PANI, PEDOT:PSS & CsPbBr_3_; scheme of optical setup; spectrum of white light source LSW-1; TEM images of Ti_3_C_2_T_X_, In_2_S_3_, and CsPbBr_3_; tabulated atom percentages measured by EDX; EDX spectra; AFM images of MXene composites; *IV* characteristics; additional photocurrent density and responsivity plots; energy-band diagrams; frequency-dependent photocurrent measurements; and tabulated figures of merit (PDF)
Bubble printing of Ti_3_C_2_T_X_ MXene on glass substrate (11x sped up) (MP4)
