# Supplementary material for: Laser-Directed Bubble Printing of MXene-Based Composites: A Simple Route to Micropatterned Photodetectors
Source: ACS Appl Mater Interfaces. 2025 Jun 23;17(26):38269–79. doi: 10.1021/acsami.5c08769 (PMC12232271; doi:10.1021/acsami.5c08769)
Supplement: Supplementary file 1 [file am5c08769_si_001.pdf]

Supporting Information:

Laser-Directed Bubble Printing of MXene-Based Composites: A Simple Route to Micropatterned Photodetectors

Marcel Herber, Bianca M. Hanly, Eric H. Hill*
Institute of Physical Chemistry, University of Hamburg, Grindelallee 117, 20146 Hamburg, Germany
The Hamburg Center for Ultrafast Imaging (CUI), Luruper Chaussee 149, 22761 Hamburg, Germany
*corresponding author: eric.hill@chemie.uni-hamburg.de

Video S1: Bubble printing of $Ti_3C_2T_X$ MXene on glass substrate, 11x sped up.

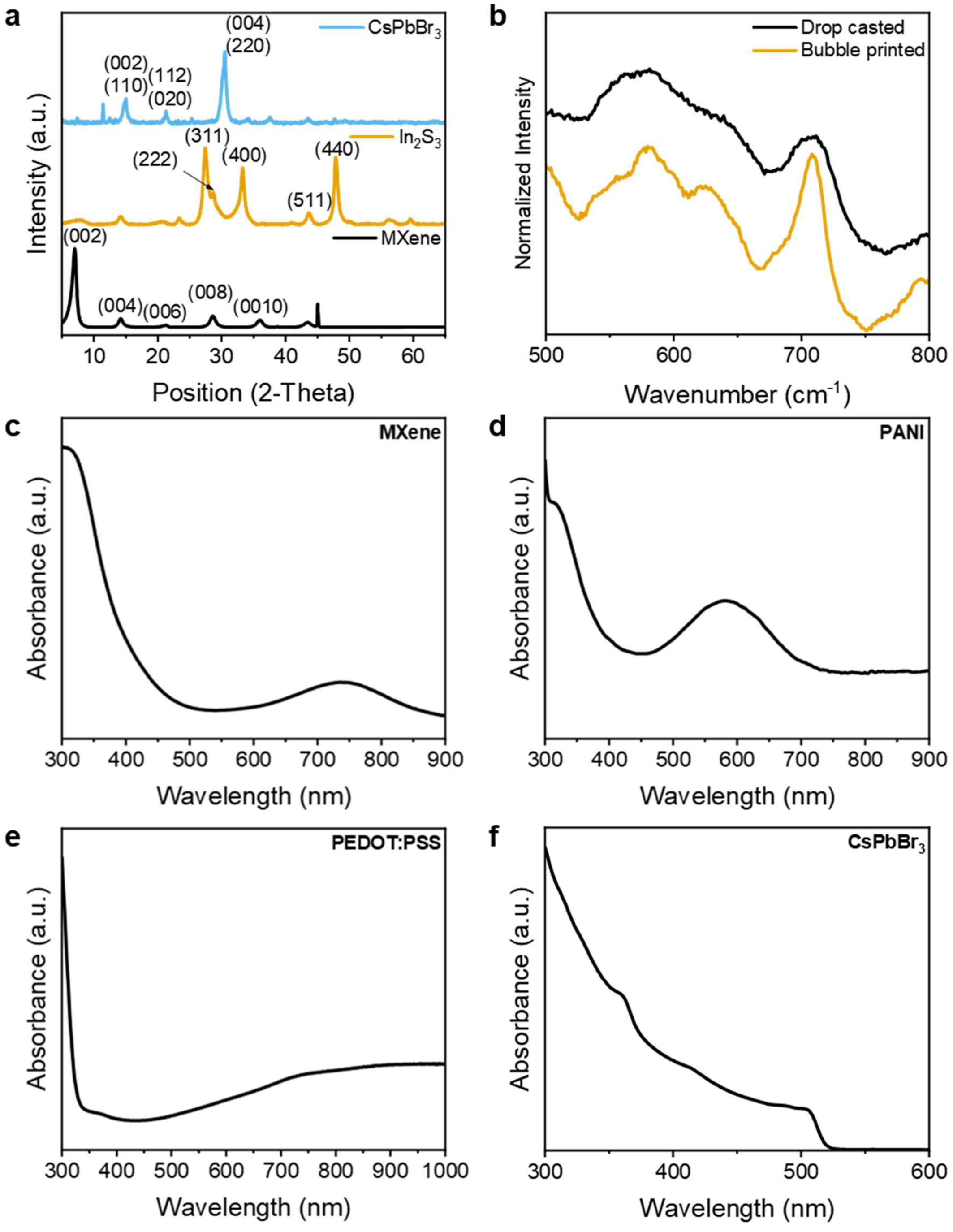

Figure S1. (a) X-ray diffraction (XRD) spectrum of $Ti_3C_2T_X$ MXene, In_2S_3 & $CsPbBr_3$ cubes; (b) Raman spectra of drop casted and bubble printed MXene; UV-Vis spectrum of (c) synthesized $Ti_3C_2T_X$ MXene, (d) PANI, (e) PEDOT:PSS and (f) $CsPbBr_3$ cubes.

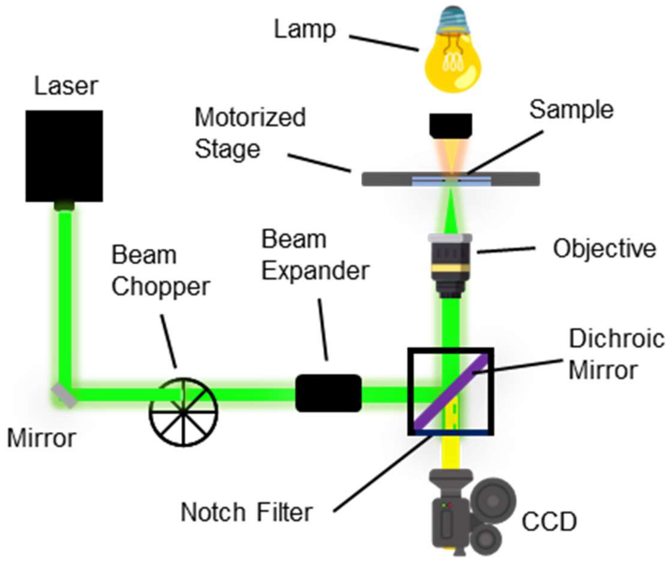

Figure S2. Scheme of the optical setup used for bubble printing.

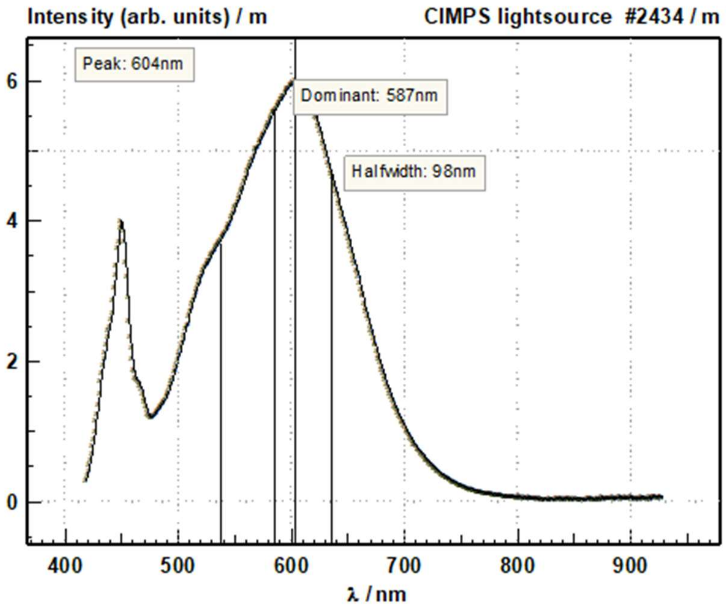

Figure S3. Spectrum of the white light source LSW-1.

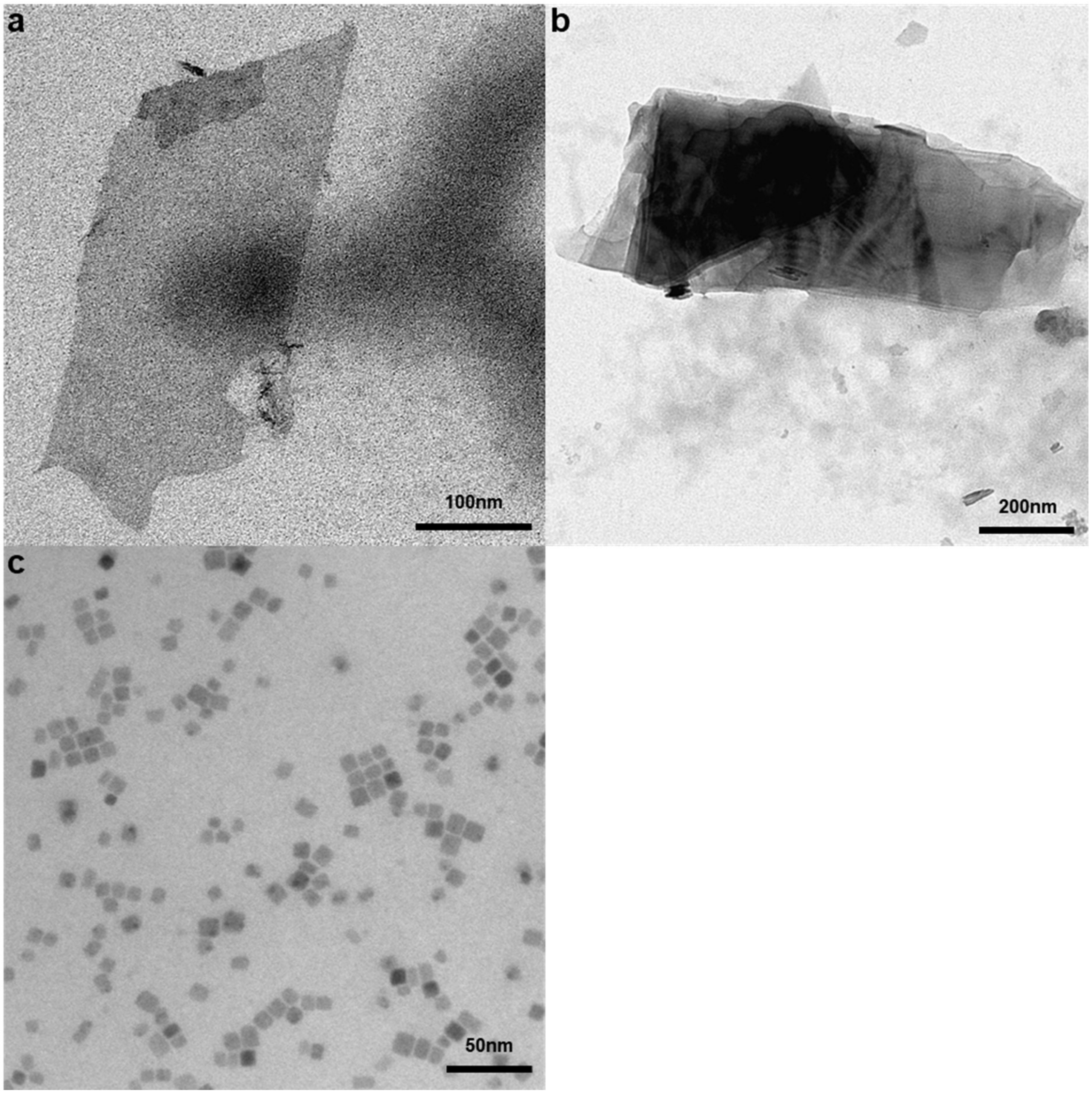

Figure S4. TEM image of (a) a MXene flake, (b) stacked In_2S_3 sheets and (c) $CsPbBr_3$ cubes.

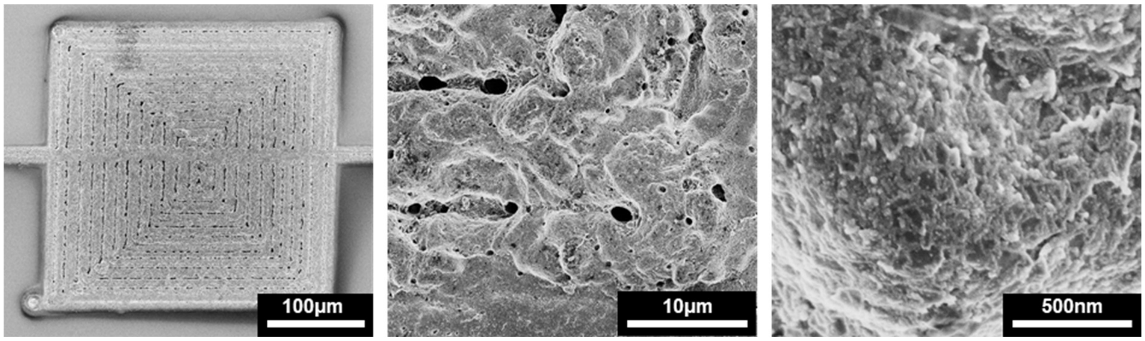

Figure S5. Scanning electron micrographs of bubble printed 1:1 MXene-In_2S_3.

Table S1. Atom percentage of various elements in different samples measured using EDX. Samples were measured on glass substrates, resulting in background signals from various elements (K, Ca, Si, O, C, Cl). K, for example, has a potential elemental overlap with In.

Element	**MXene**	**9:1 MXene-In_2S_3**	**1:1 MXene-In_2S_3**	**MXene-CuS**	**MXene-$CsPbBr_3$**
	Atom %				
Ti	36.39	42.26	29.52	27.41	23.82
C	51.77	46.92	50.29	53.74	57.67
F	11.84	9.58	8.29	15.87	17.40
In	-	0.86	6.26	-	-
S	-	0.38	5.64	1.08	-
Cu	-	-	-	1.90	-
Cs	-	-	-	-	0.34
Pb	-	-	-	-	0.44
Br	-	-	-	-	0.33

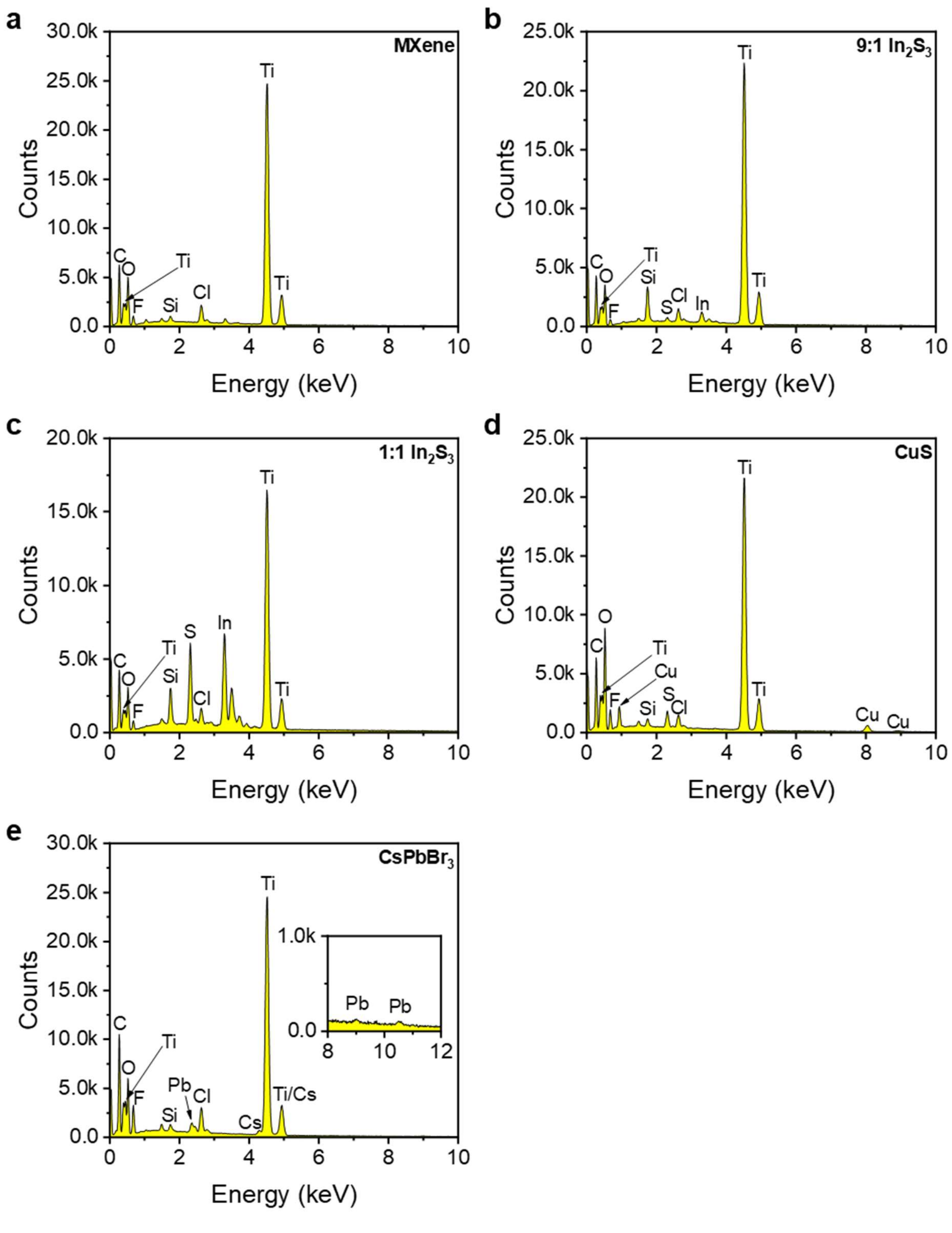

Figure S6. EDX spectra of (a) MXene, (b) 9:1 MXene-In_2S_3, (c) 1:1 MXene-In_2S_3, (d) MXene-CuS and (e) MXene-$CsPbBr_3$. Samples were measured on glass.

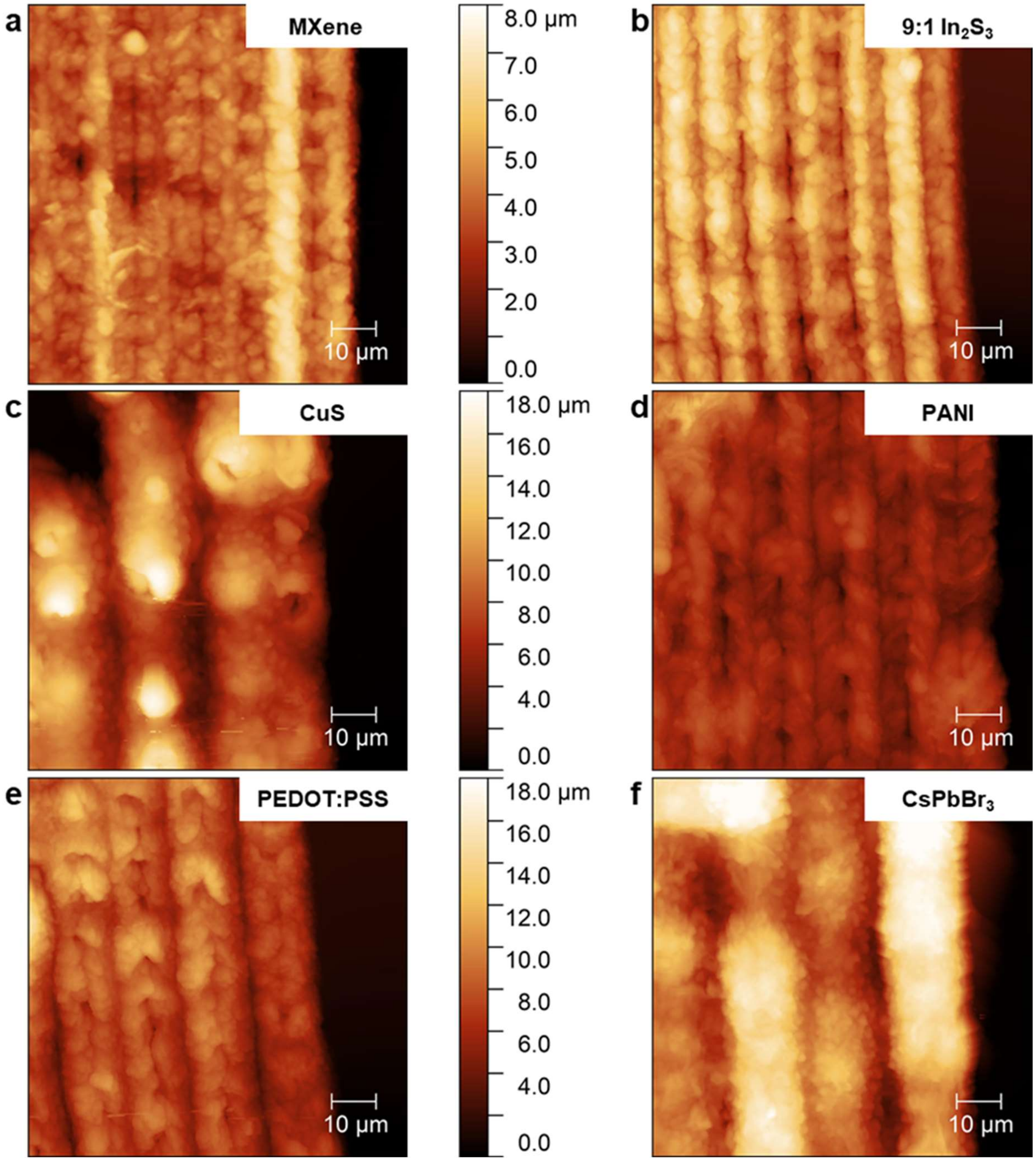

Figure S7. Atomic force microscopy images of bubble printed (a) MXene, (b) 9:1 MXene-In_2S_3, (c) MXene-CuS, (d) MXene-PANI, (e) MXene-PEDOT:PSS and (f) MXene-$CsPbBr_3$.

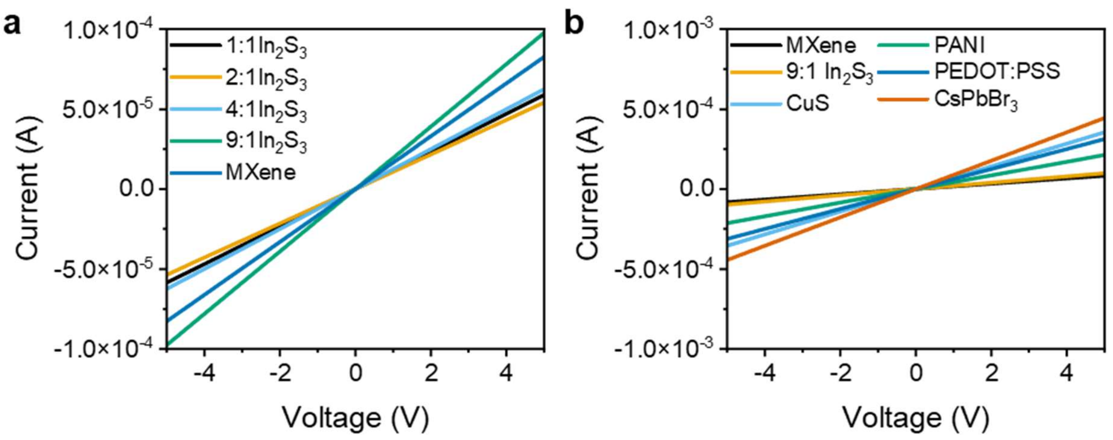

Figure S8. IV curves of (a) bubble printed MXene-In_2S_3 patterns with different ratios of MXene to In_2S_3 and (b) different bubble printed MXene-composite patterns.

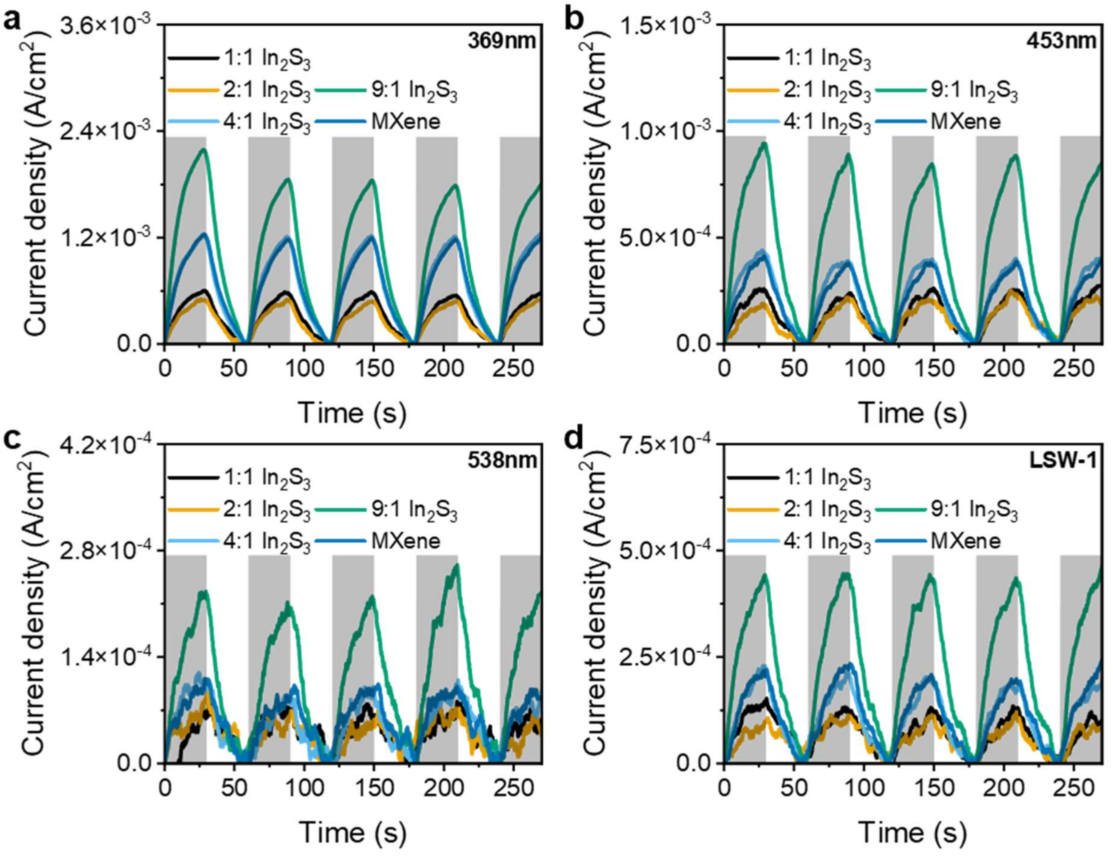

Figure S9. Photocurrent density responses of MXene-In_2S_3 patterns with different ratios under (a) 369 nm, (b) 453 nm, (c) 538 nm and (d) white light irradiation. The gray shaded regions indicate light on cycles in the measurements with an intensity of 100%. **Figure S10** shows the current density at different light intensities.

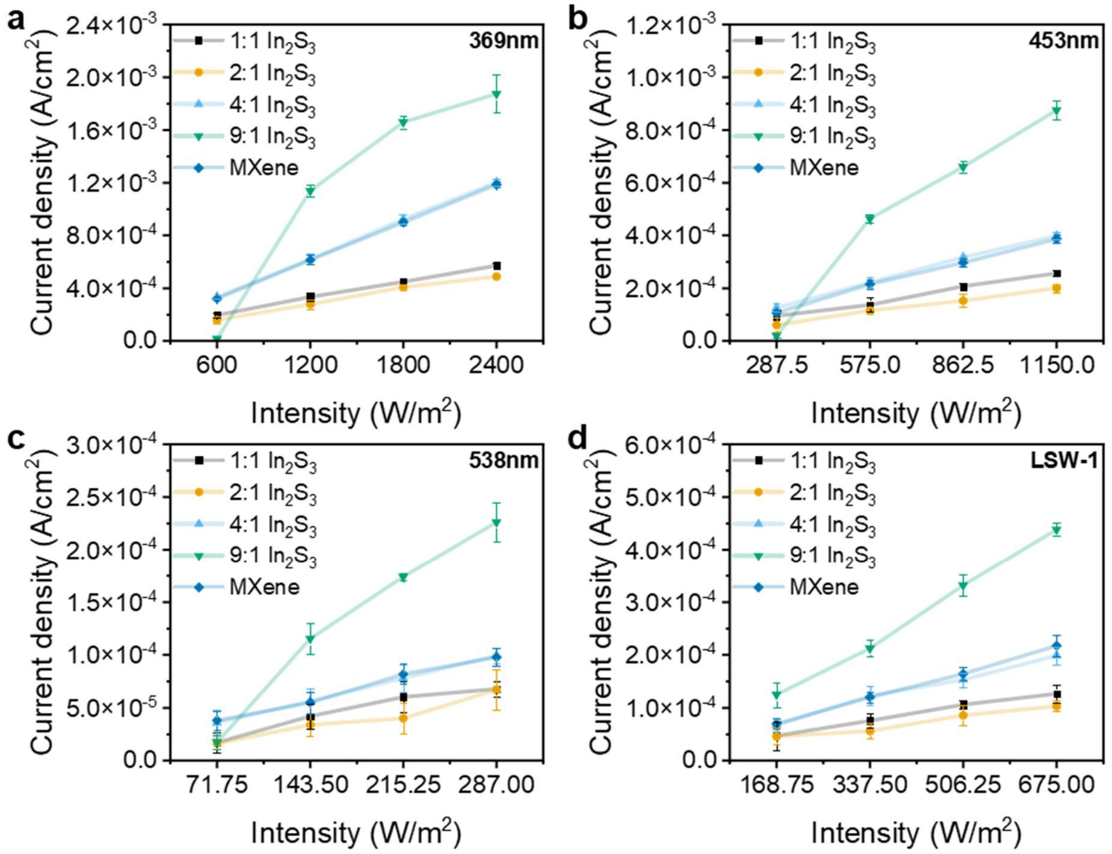

Figure S10. Photocurrent density versus light intensity of different rations of MXene-In_2S_3 for (a) a 369 nm LED, (b) a 453 nm LED, (c) a 538 nm LED and (d) white light irradiation.

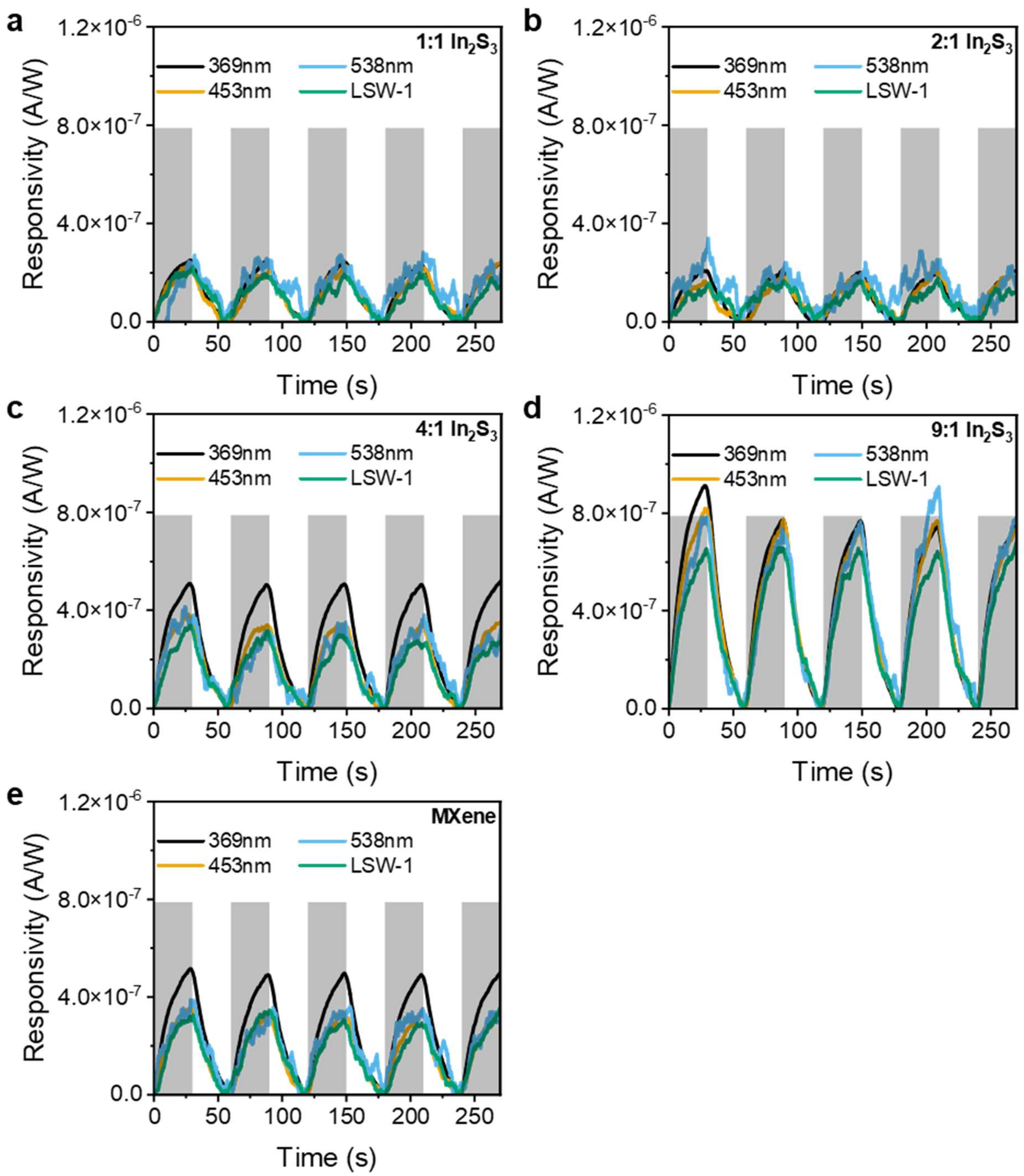

Figure S11. Photoresponse in responsivity for (a) 1:1 MXene:In_2S_3, (b) 2:1 MXene:In_2S_3, (c) 4:1 MXene:In_2S_3, (d) 9:1 MXene:In_2S_3 and (d) MXene. The gray shaded regions indicate light on cycles in the measurements.

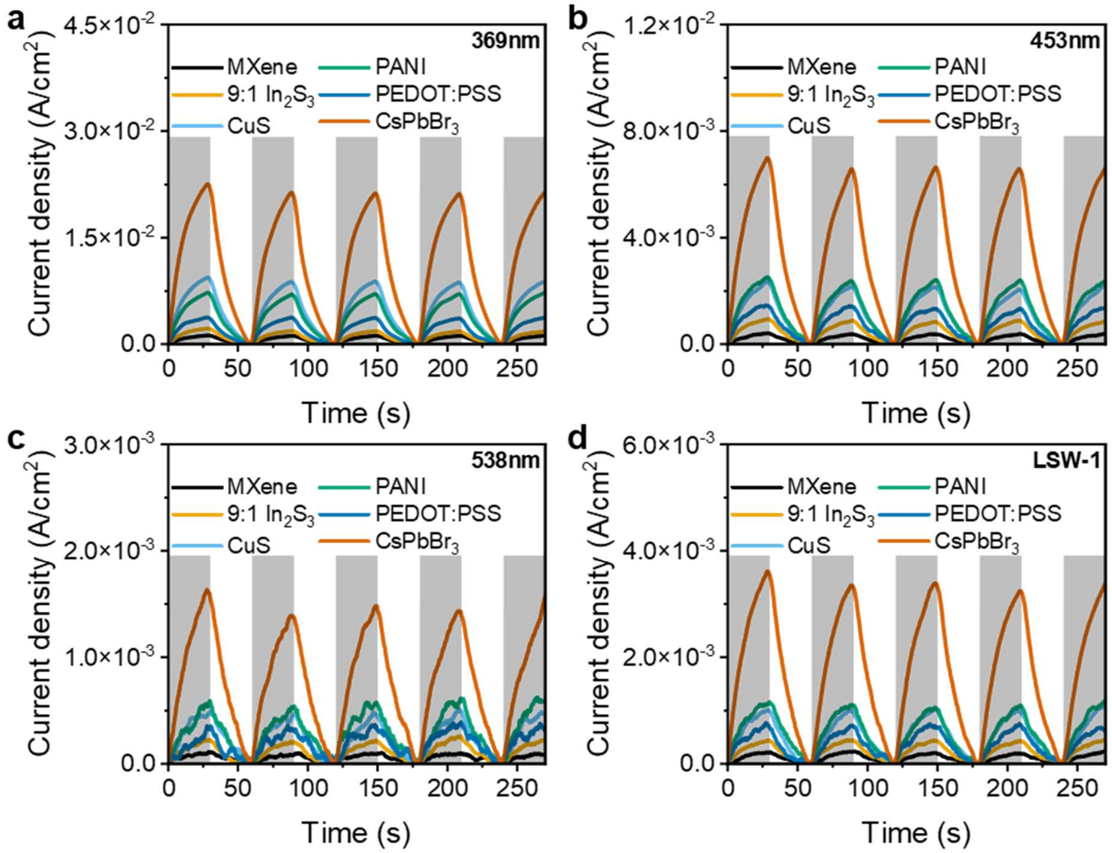

Figure S12. Photocurrent density responses of different MXene-composite patterns under (a) 369 nm, (b) 453 nm, (c) 538 nm and (d) white light irradiation. The gray shaded regions indicate light on cycles in the measurements with an intensity of 100%. **Figure S13** shows the current density at different light intensities.

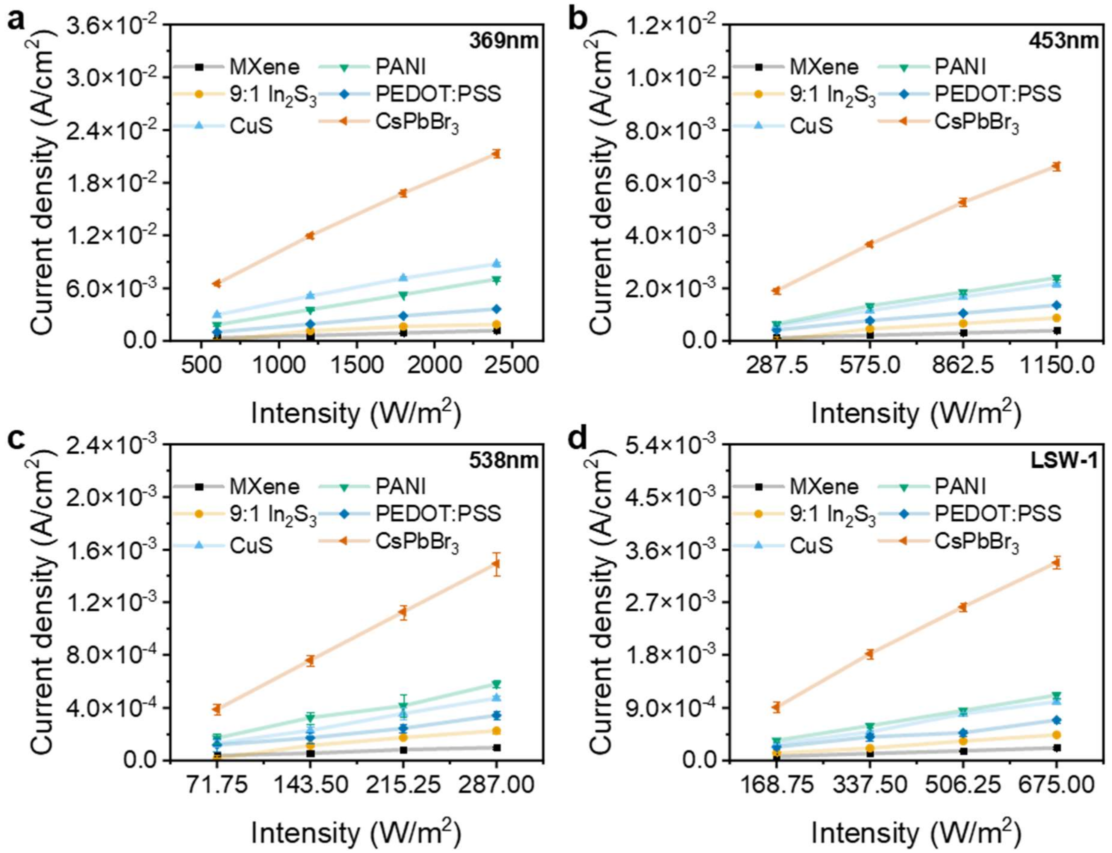

Figure S13. Photocurrent density versus light intensity of different MXene-composites for (a) a 369 nm LED, (b) a 453 nm LED, (c) a 538 nm LED and (d) white light irradiation.

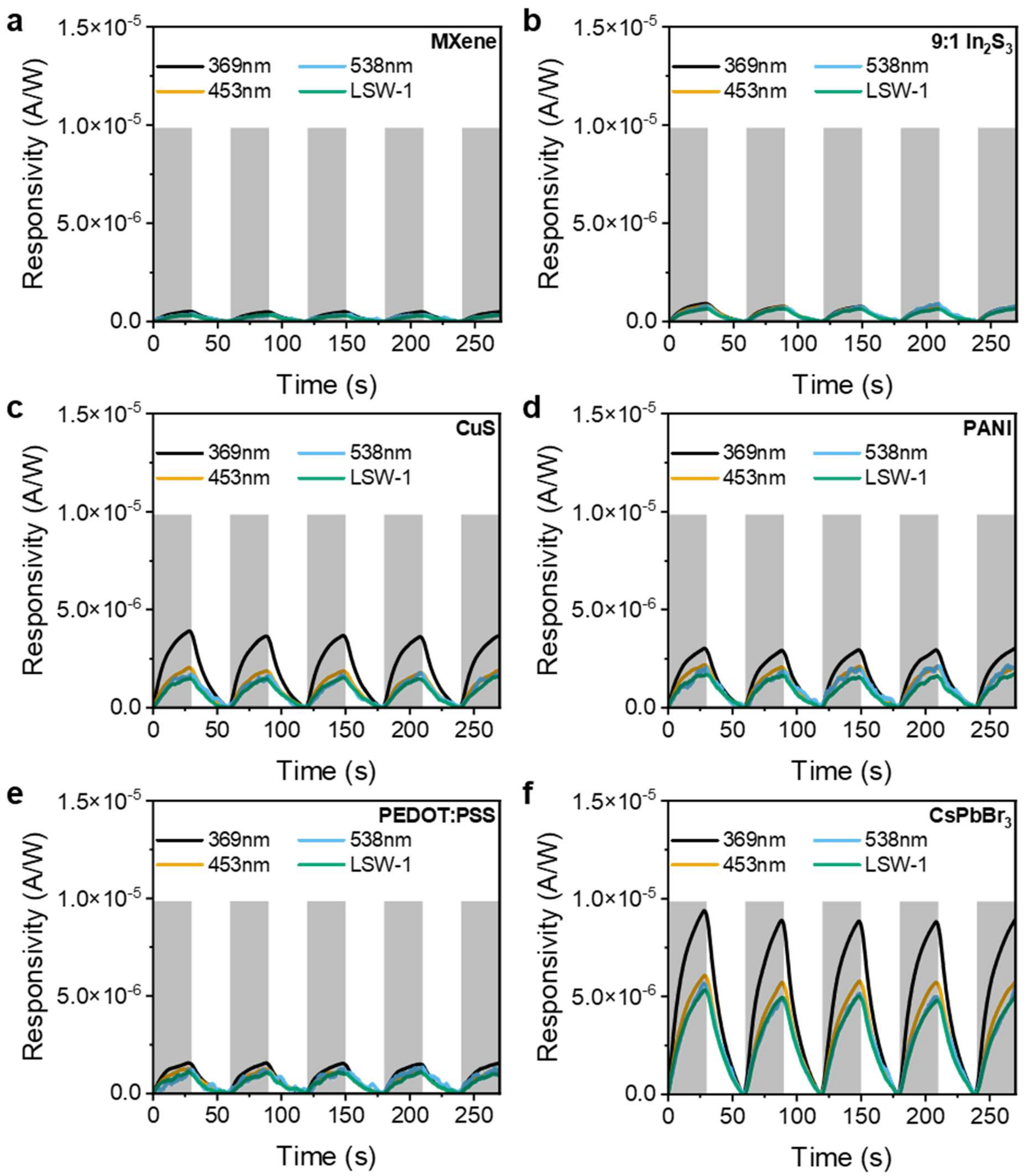

Figure S14. Photoresponse in responsivity for (a) MXene, (b) 9:1 MXene-In_2S_3, (c) MXene-CuS, (d) MXene-PANI, (e) MXene-PEDOT:PSS and (f) MXene-$CsPbBr_3$. The gray shaded regions indicate light on cycles in the measurements.

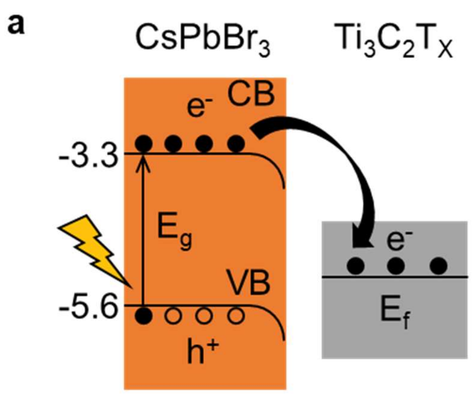

Figure S15. The energy-band diagrams of (a) MXene-$CsPbBr_3$ and (b) MXene-In_2S_3 under light illumination.

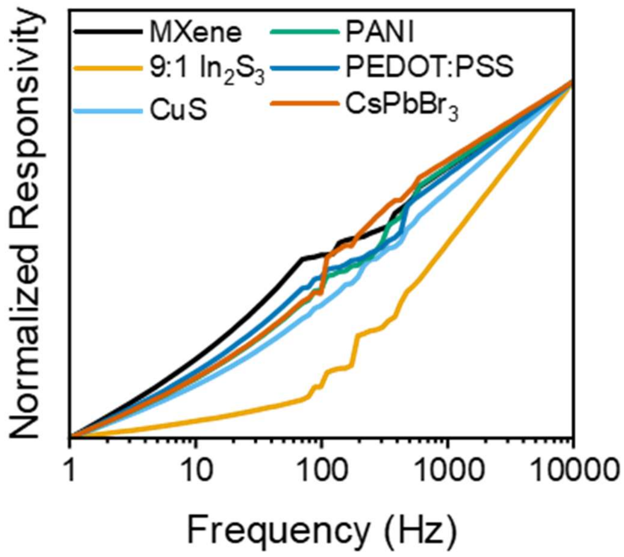

Figure S16. IMPS (intensity modulated photocurrent spectroscopy) curves of different bubble printed MXene-composite patterns with white light.

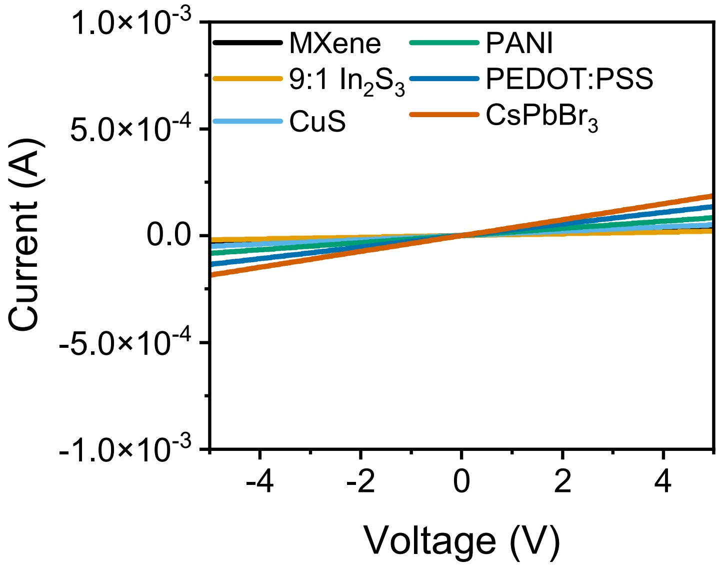

Figure S17. IV curves of different bubble printed MXene-composite patterns after 62 days in ambient air.

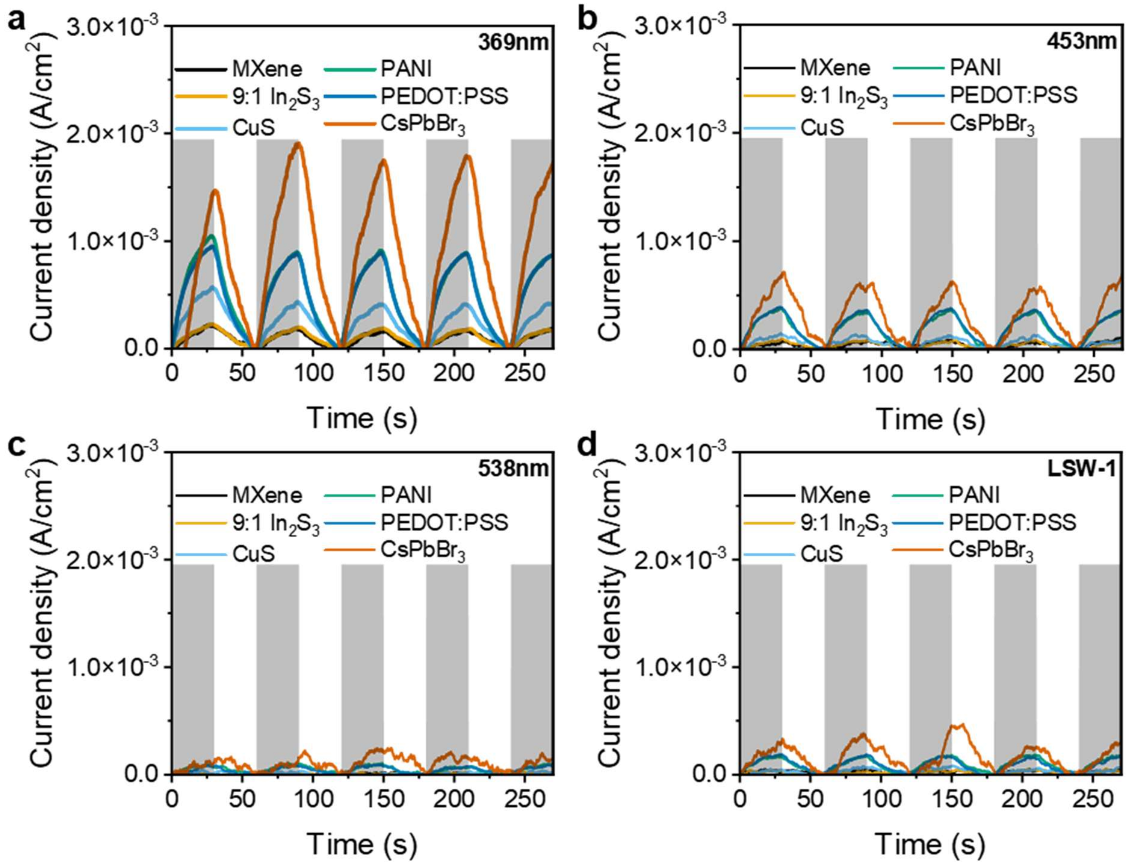

Figure S18. Photocurrent density responses of different MXene-composite patterns under (a) 369 nm, (b) 453 nm, (c) 538 nm and (d) white light irradiation after 62 days in ambient air. The gray shaded regions indicate light on cycles in the measurements with an intensity of 100%.

Table S2. Figures of Merit of different MXene-In_2S_3 ratios and different light sources with an intensity of 100%, with 5 V applied voltage.

Figure of Merit		**1:1 In_2S_3**	**2:1 In_2S_3**	**4:1 In_2S_3**	**9:1 In_2S_3**	**MXene**
R [µA/W]	369 nm	0.24	0.20	0.50	0.78	0.49
NEP [W]		131	159	60.2	30.7	52.7
D* [Jones] × 10^2		9.65	7.92	20.9	41.0	23.9
R [µA/W]	453 nm	0.22	0.17	0.35	0.76	0.34
NEP [W]		150	201	93.4	34.1	83.7
D* [Jones] × 10^2		9.05	6.76	14.5	39.8	16.2
R [µA/W]	538 nm	0.24	0.23	0.35	0.79	0.34
NEP [W]		156	165	103	36.3	91.0
D* [Jones] × 10^2		9.56	9.06	14.5	41.2	16.4
R [µA/W]	LSW-1	0.19	0.15	0.30	0.65	0.32
NEP [W]		684	721	451	159	398
D* [Jones] × 10^2		4.07	3.85	6.16	17.5	6.99

Table S3. Figures of Merit of different MXene-composites and different light sources with an intensity of 100%, with 5 V applied voltage.

Figure of Merit		**MXene**	**9:1 In_2S_3**	**CuS**	**PANI**	**PEDOT:PSS**	**$CsPbBr_3$**
R [µA/W]	369 nm	0.49	0.78	3.66	2.93	1.52	8.88
NEP [W]		52.7	30.7	3.45	5.55	8.86	1.27
D* [Jones] × 10^3		2.39	4.10	36.5	22.7	14.2	99.1
R [µA/W]	453 nm	0.34	0.76	1.88	2.08	1.18	5.77
NEP [W]		83.7	34.1	7.24	8.42	12.3	2.11
D* [Jones] × 10^3		1.62	3.98	18.7	16.1	11.0	64.4
R [µA/W]	538 nm	0.34	0.79	1.65	2.03	1.20	5.20
NEP [W]		91.0	36.3	9.07	9.55	13.4	2.58
D* [Jones] × 10^3		1.64	4.12	16.5	15.7	11.2	58.0
R [µA/W]	LSW-1	0.32	0.65	1.49	1.65	1.03	5.00
NEP [W]		398	159	18.7	21.7	28.9	4.98
D* [Jones] × 10^3		0.70	1.75	14.9	12.8	9.61	55.8
